# Changes in sediment greenhouse gases production dynamics in an estuarine wetland following invasion by *Spartina alterniflora*

**DOI:** 10.3389/fmicb.2024.1420924

**Published:** 2024-07-12

**Authors:** Yongcan Jiang, Yinlong Shao, Jiafang Huang, Yunling Du, Yu Wen, Hong Tang, Jianming Xu, Dengzhou Gao, Xianbiao Lin, Dongyao Sun

**Affiliations:** ^1^Power China Huadong Engineering Corporation Ltd., Hangzhou, Zhejiang Province, China; ^2^College of Environmental and Resource Sciences, Institute of Soil and Water Resources and Environmental Science, Zhejiang University, Hangzhou, Zhejiang Province, China; ^3^Institute of Geography, Fujian Normal University, Fuzhou, China; ^4^Frontiers Science Center for Deep Ocean Multispheres and Earth System, Key Laboratory of Marine Chemistry Theory and Technology, Ministry of Education, Ocean University of China, Qingdao, China; ^5^School of Geography Science and Geomatics Engineering, Suzhou University of Science and Technology, Suzhou, China

**Keywords:** greenhouse gases, *Spartina alterniflora* invasion, estuarine and coastal wetland, sediment, CH_4_, CO_2_, N_2_O

## Abstract

Invasive *Spartina alterniflora* (*S. alterniflora*) has significant impacts on sediment biogeochemical cycling in the tidal wetlands of estuaries and coasts. However, the impact of exotic *Spartina alterniflora* invasion on greenhouse gases (GHGs) production dynamics in sediments remain limited. Here, we investigated the dynamics of sediment physicochemical properties, GHGs production rates, and microbial gene abundances in a native *Cyperus malacensis* habitat and three invasive *S. alterniflora* habitats (6-, 10-, and 14-year) in the Minjiang River Estuary, China. The methane (CH_4_), carbon dioxide (CO_2_), and nitrous oxide (N_2_O) production rates varied both spatially and seasonally, while microbial gene abundances (bacterial and fungal gene abundances) and organic matter (TOC and TN) only varied spatially. GHGs production rates were also characterized by higher values in surface sediment (0–10 cm) compared to subsurface sediment (10–20 cm) and by seasonal variations with higher values in summer than in winter. *S. alterniflora* invasion can significantly increase CH_4_ and CO_2_ production rates, organic matter, and microbial gene abundances (*p* < 0.05). Temperature, organic matter and microbial gene abundances were the most dominating factor controlling the spatio-temporal variations of CH_4_ and CO_2_ production rates. Overall, our findings highlighted the significant role of *S. alterniflora* invasion in regulating GHGs production rates in coastal wetland sediments and provided fundamental data for estimating GHGs emissions and carbon sequestration in the complex tidal wetlands.

## 1 Introduction

In 1979, *Spartina alterniflora* (*S. alterniflora*) was intentionally introduced to the estuarine and coastal wetlands of China with the objective of promoting siltation and coastal protection (Lu and Zhang, 2013). Over the last forty years, it has rapidly invaded the bare flats, salt marshes, and mangroves along coastal zone, and has expanded its presence to cover ∼344.51 km^2^ of land (Wang and Lin, 2023; Wu et al., 2024). *S. alterniflora* is a salt-tolerant plant, which can effectively protect coastlines, withstand typhoons, reduce pollution and provide habitats for many benthic species (Cagle et al., 2020; Wang et al., 2022). Nevertheless, its rapid growth comes with certain disadvantages, including obstructing waterways, biodiversity loss and posing a threat to native communities (Liao et al., 2008; Wu et al., 2024). Meanwhile, *S. alterniflora* invasion may alter ecosystem structure and material cycling processes, which in turn have a substantial impact on sediment biogeochemical cycles and ultimately altering ecosystem functions and services (Li et al., 2009). Hence, the impact of *S. alterniflora* invasion on the biogeochemical cycles of carbon (C) and nitrogen (N) in coastal sediments has garnered significant attentions.

Previous studies have shown that *S. alterniflora*, a C4 plant, can substantially increase sediment C and N stocks after invading the bare flat and C3 plants (Zhang et al., 2010a; Feng et al., 2017). This invasive plant characterized by the C4 photosynthetic pathway, has a higher net primary productivity than native plants such as *Phragmites australis*, *Suaeda salsa*, and *Cyperus malacensis* (*C. malacensis*) thereby increasing the storage of C and N in biomass (Zhang et al., 2010a). For instance, *S. alterniflora* invasion has been shown to significantly elevate sediment organic carbon, total nitrogen, and total phosphorus levels compared to nearby bare flats (Wang et al., 2016). This is mainly because *S. alterniflora* invasion can increase the organic matter contents in sediment by decomposing plant litter and roots, promoting sedimentation, and adsorbing organic matter (Gao et al., 2017). Under normal circumstances, *S. alterniflora* invasion can increase the abundances and diversity of microorganisms in sediments of bare flat and native C3 plants (Lin et al., 2022). Nevertheless, it can also reduce the species diversity and abundance or density of benthic animals of the native herbs (*Phragmites australis* and *Suaeda salsa*) (Yu et al., 2022). In addition, *S. alterniflora* invasion can also speed up the rates of organic carbon sequestration and N conversion, enhance N mineralization rates, and boost N-fixation rates (Chen et al., 2016; He et al., 2019). Consequently, it is commonly found that *S. alterniflora* invasion in bare flats and native C3 plants can effectively enhance C and N storage in sediment, subsequently expediting sediment C and N recycling.

With the intensification of *S. alterniflora* spreading in tidal wetlands, its impact on the environment is becoming increasingly apparent. Among these impacts, the impact of *S. alterniflora* invasion on GHGs production in tidal wetlands has attracted considerable attention. Although numerous studies have been carried out to examine sediment GHGs emissions in *S. alterniflora* salt marsh ecosystems (Cheng et al., 2010; Tong et al., 2012; Yin et al., 2015; Yuan et al., 2015), there is little consensus on the impact of *S. alterniflora* invasion on sediment GHGs emissions. For instance, *S. alterniflora* invasion can lead to a notable increase in methane (CH_4_) emissions after invaded bare flats or C3 plants due to its higher plant biomass (Cheng et al., 2010; Zhang et al., 2010b; Tong et al., 2012; Yuan et al., 2014). In contrast, *S. alterniflora* invasion was reported to decrease in nitrous oxide (N_2_O) emissions after invaded bare flats or C3 plants (Yuan et al., 2015; Gao et al., 2019), and to reduce carbon dioxide (CO_2_) emissions after invaded mangrove wetland sediment in southeastern China (Gao et al., 2018). Also, a previous study found that there were no significant differences in GHGs emissions between *S. alterniflora* and *Phragmites australis* stands in a New England marsh. In addition, *S. alterniflora* invasion was reported to enhance CO_2_ while reduce CH_4_ emissions after invad*ed* native *P. australis* marshes in Yancheng National Nature Reserve, China (Xu et al., 2014). This inconsistency implies that suggests that the impact of *S. alterniflora* encroachment on the carbon and nitrogen cycles within sedimentary ecosystems is multifaceted. As the rapid expansion of *S. alterniflora* in China’s coastal wetlands, it has become critically imperative to reveal the sediment GHGs emission from wetland as well as its driven factors according to the degree of *S. alterniflora* invasion.

In summary, despite numerous previous studies on the response of sediment GHGs emissions to *S. alterniflora* invasion in bare flats, C3 plants or mangrove (Tong et al., 2012; Yuan et al., 2014; Xiang et al., 2015; Gao et al., 2018; Bu et al., 2019; He et al., 2021), systematic research on the role of *S. alterniflora* in regulating the spatio-temporal variations of GHGs and influencing mechanism in *C. malacensis* wetland following invasion chronosequences remains unclear. Here, sediment GHGs production rates, physicochemical properties and microbial gene abundances were measured following *S. alterniflora* invasion in a chronosequence of 6-, 10-, and 14-year-old by comparing with native *C. malacensis* in the Minjiang River Estuary, China. The aims of our research were (1) to explore spatio-temporal variations of sediment GHGs production rates in *C. malacensis* wetland invaded by *S. alterniflora*; (2) to elucidate the key environmental factors regulating sediment GHGs production rates in *C. malacensis* wetland invaded by *S. alterniflora*; (3) to offer fundamental data for evaluating the ecological and environmental impacts resulting from *S. alterniflora* invasion into *C. malacensis* ecosystems.

## 2 Materials and methods

### 2.1 Research location and sediment collection

**FIGURE 1:**
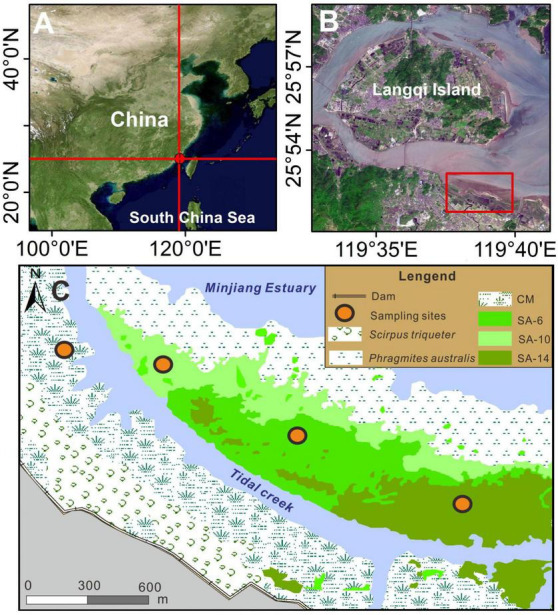
Study area **(A,B)** and sampling locations **(C)**, which was modified according to a previous study (Gao et al., 2017). The CM denotes *C. malacensis* habitat, and the SA-6, SA-10, and SA-14 denote *S. alterniflora* habitats for 2–6 years, 6–10 years, and 10–14 years, respectively.

Shanyutan (26.01–26.06°N, 119.57–119.69°E) is the largest intertidal wetlands in the Minjiang Estuary, China (Figures 1A,B). It is characterized by a typical subtropical monsoon climate with an annual mean temperature of ∼19°C and annual mean precipitation of ∼1300 mm (Mou et al., 2018). The commonest native plants in this estuarine wetlands are *Scirpus triqueter*, *C. malacensis*, and *Phragmites australis*, which mainly grow in low, middle, and high intertidal zones, respectively (Gao et al., 2017). In 2002, the introduction of *S. alterniflora* to the study area led to its rapid expansion and colonization of the native *C. malacensis* habitat in the middle intertidal zone over the following two decades (Mou et al., 2014). Superimposing and analyzing Landsat 8 (2014), SPOT5 (2010), and aerial (2006) images were employed to identify sampling locations showcasing varying chronosequences of invasive *S. alterniflora* (Jin et al., 2017). The sampling locations consisted of a native habitat (CM, *C. malacensis*) and three invasive *S. alterniflora* habitats that took over the native habitat during different time periods: SA-6 (2010–2014), SA-10 (2006–2010), and SA-14 (2002–2006) (Figure 1C). The four habitats mentioned above were selected as sampling points, representing varying degrees of invasion by *S. alterniflora*. The sampling locations (CM, SA-6, SA-10, and SA-14) were located in the middle intertidal zone, which had similar sediment and hydrodynamic properties prior to the invasion of *S. alternifolia*. Within each sampling habitat, three sediment samples were randomly collected at two depths (0–10 cm and 10–20 cm) using a stainless steel sediment cylinder with a diameter of 10 cm in July and December 2016. We transported all samples to the laboratory on ice. Three parallel sediments from each site were mixed thoroughly under anaerobic conditions in the laboratory. Sediment were divided into three parts. One part was frozen at −20°C for physical and chemical parameter analysis, the second part was stored at 4°C to determine GHGs production rates, and the remaining part was kept at −80°C for microbiological analysis.

### 2.2 Analysis of sediment physicochemical characteristics

The portable thermometer was used to measure soil temperatures. Sediment pH and electrical conductivity (EC) were determined by the handheld pH (IQ Scientific Instruments, USA) and EC tester (Spectrum Technologies Inc., USA), respectively. The oven-drying (fresh sediment at 60°C to a constant weight) and cutting-ring methods were used to analyze sediment moisture and density, respectively. Total organic carbon (TOC) and total nitrogen (TN) contents in sediments were measured based on a CHNS analyzer (Vario EL, Elementar, Germany) after pretreatment of the sediment with 5% HCl to remove inorganic carbon with (Lin and Lin, 2022). The potassium chloride solution (KCl, purged with N_2_ for 15 min) of 2 M was applied to extract sediment ammonium (NH_4_^+^), nitrate (NO_3_^–^) and nitrite (NO_2_^–^), and their contents were measured by an automatic injection analyzer (Skalar SAN++, the Netherlands) (Huang et al., 2021). Sediment sulfide (S^2–^) contents were assessed via methylene blue spectrophotometry (Lin et al., 2017).

### 2.3 Determination of GHGs production rates and their temperature sensitivity

In this study, all samples were incubated anaerobically at *in situ* temperature in the dark for 24 h. In brief, three brown glass bottles (250 mL) were prepared for each sediment sample. 10 g fresh sediment was placed in each bottle. Anaerobic glass bottles and three sediment-free controls were flushed with N_2_ for 5 min and then sealed with airtight butyl rubber stopper for 24 h to allow the accumulation of GHGs (Lang et al., 2011). Then, ∼12 mL of headspace gas samples were collected from each bottle (after vigorous shaking) using a syringe, and then stored in 12 mL evacuated vials. CO_2_, CH_4_, and N_2_O concentrations were determined via gas chromatography Shimadzu GC-2014B using Porapak-N and HayeSep-D and Molecular Sieve MS13 columns and equipped with TCD, FID, and ECD detectors (Morrissey and Franklin, 2015). GHGs production rates were calculated based on the accumulation of gas production within the incubation period using linear regression. To obtain the overall GHGs production rate, three GHGs rates were converted to a common unit (CO_2_-equivalent) and added together using the Global Warming Potential over a 100-year timeframe of 28 for CH_4_ and 273 for N_2_O set by the Intergovernmental Panel on Climate Change (IPCC, 2021).

**FIGURE 2:**
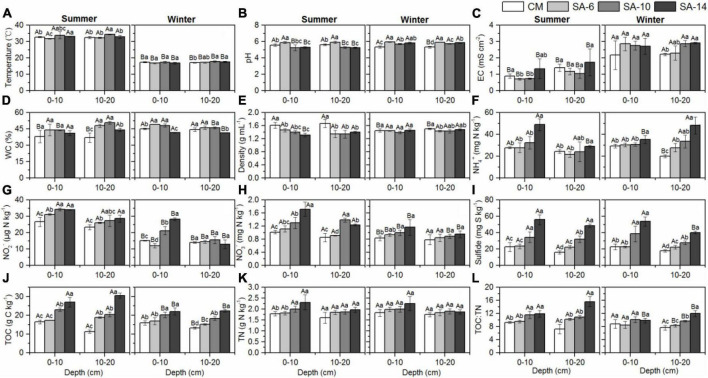
The effects of invasive *S. alternifolia* on sediment physicochemical properties (Mean ± SD) along four habitats. Sediment physicochemical properties including temperature **(A)**, pH **(B)**, electrical conductivity **(C)**, water content **(D)**, sediment bulk density **(E)**, exchangeable inorganic N **(F–H)**, sulfide **(I)**, TOC **(J)**, TN **(K)**, and TOC:TN **(L)** were measured in summer and winter. The CM denotes *C. malacensis* habitat, and the SA-6, SA-10, and SA-14 denote *S. alterniflora* habitats for 2–6, 6–10, and 10–14 years, respectively. Different lowercase letters are used to denote statistical differences (*p* < 0.05) across four habitats in the same depth, while upper case letters are used to denote significant differences (*p* < 0.05) between the summer and winter in each habitat.

To determine the temperature response of sediment GHGs production rates, temperature sensitivity (Q_10_) was estimated based on the modified van’t Hoff’s equation, which is shown as follows (Equation 1):


(1) $$Q_{10}={\left(R_{T_{s}}/R_{T_{w}}\right)}^{[\left(10/\left(T_{s}-T_{w}\right)\right]}$$

where *R*_*T1*_ and *R*_*T2*_ are sediment GHGs production rates in summer and winter, respectively. *T*_*s*_ and *T*_*w*_ are incubation temperature in summer and winter, respectively (Figure 2A).

### 2.4 Microbial analysis

DNA extraction from sediment samples was carried out using the FastDNA spin kit for sediment (MP Biomedical, USA) according to the manufacturer’s instructions. The concentration and purity of the extracted DNA were assessed using a NanoDrop spectrophotometer (ND-2000C, Thermo Scientific, USA). The size and quality of DNA fragments were evaluated by 1% agarose gel electrophoresis. To quantify the gene abundances (Bacterial 16S rRNA, Fungal) in the microbial communities, qPCR assays (Applied Biosystems, USA) were performed on an ABI 7500 Fast real-time qPCR system (Applied Biosystems, USA). Abundance of bacterial 16S rRNA genes was quantified via Quantitative PCR using 341F and 519R primers (Muyzer et al., 1993). Fungal genes abundance was determined using PCR primers SSU0817F and 1196R (Borneman and Hartin, 2000).

### 2.5 Statistical analysis

All data analysis was performed using the SPSS 19.0 and Origin 2021. Data were assessed for normal distribution and the requirement for any potential transformations prior to conducting the statistical analysis. The significant differences of sediment physicochemical properties, GHGs production, and microbial abundances between different habitats were analyzed by one-way ANOVA, Turkey test (*p* < 0.05, equal variances assumed). The significant differences of sediment physicochemical properties, GHGs production rates, and microbial gene abundances between summer and winter, and between surface and subsurface sediments were both tested by independent-samples t-test (*p* < 0.05). Linear regression analyses and the Pearson correlation test (two-tailed, *p* < 0.05) were used to examine the relationships between variables. Multiple stepwise regression analyses were employed to investigate the relationships between GHGs production rates and various environmental variables.

## 3 Results

### 3.1 Sediment properties

Sediment temperature ranged from 16.80 to 33.83°C, with summer temperatures (32.95 ± 1.09°C) being significantly higher than those in winter (17.28 ± 0.47°C) (Figure 2A). During summer, sediment pH ranged from 5.18 to 5.91, with the highest levels observed in SA-6 habitats, followed by the CM habitats, and the lowest levels recorded in the SA-14 and SA-10 habitats. In winter, sediment pH was notably higher in *S. alterniflora* habitats (SA-14, SA-6, and SA-10) compared to the *C. malacensis* habitat (Figure 2B). Sediment electrical conductivity (EC) ranged from 0.70 to 1.73 mS cm^–2^ and 2.17 to 2.91 mS cm^–2^ in summer and winter, respectively. The highest values were consistently observed in the SA-14 habitats (Figure 2C). Sediment water contents ranged from 32.09% to 51.74% and from 41.24% to 49.63% in summer and winter, respectively (Figure 2D). Sediment bulk density were in a range of 1.22-1.74 mg mL^–1^ and 1.36-1.52 mg mL^–1^ in summer and winter, respectively (Figure 2E). In general, sediment sulfide, NH_4_^+^, NO_3_^–^ and NO_2_^–^, TN, TOC, and TOC:TN increased with the invasion degree of *S. alterniflora* (Figures 2F–L). In addition, sediment TN, NH_4_^+^, NO_3_^–^ and NO_2_^–^ contents at the surface (0-10 cm) were higher than those at the subsurface (10-20 cm), except for winter NH_4_^+^ in SA-10 and SA-14 habitats (Figures 2F–H, L). No obvious differences in temperature, pH, water contents, and density were observed between 0-10 cm and 10-20 cm depth (Figures 2A, B, D, E).

### 3.2 Sediment GHGs production rates

**FIGURE 3:**
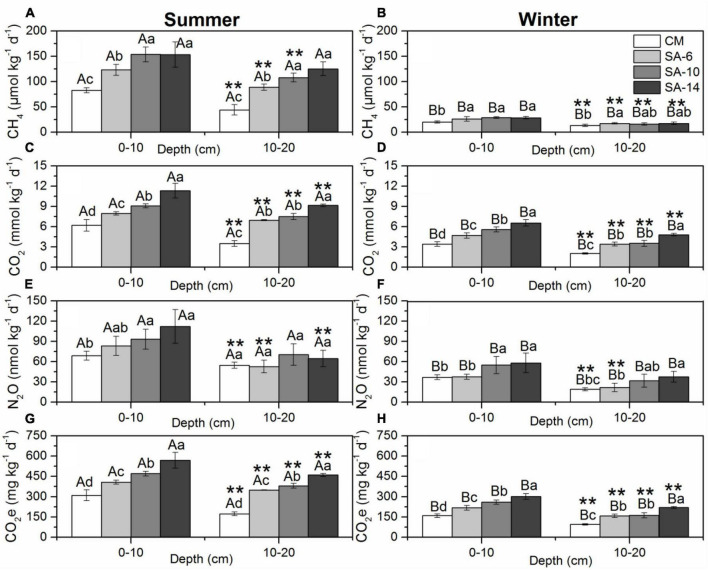
The effects of invasive *S. alternifolia* on sediment GHGs production rates (Mean ± SD) along four habitats. Sediment GHGs production rates including CH4 production rates **(A,B)**, CO_2_ production rates **(C,D)**, N_2_O production rates **(E,F)**, and total GHG production rates **(G,H)** were measured in summer and winter. The CM denotes *C. malacensis* habitat, and the SA-6, SA-10, and SA-14 denote *S. alterniflora* habitats for 2–6 years, 6–10 years, and 10–14 years, respectively. Different lowercase letters are used to denote statistical differences (*p* < 0.05) across four habitats in the same depth, while upper case letters are used to denote significant differences (*p* < 0.05) between the summer and winter in each habitat. ** are used to denote significant differences (*p* < 0.05) between surface and subsurface sediments.

Sediment GHGs production rates varied both spatially and seasonally. Spatially, GHGs production rates generally increased from CM to SA-6 to SA-10 then to SA-14, and all the values in 0-10 cm sediment layer were higher than those in 10-20 cm sediment layer in these four habitats. Seasonally, all rates were higher in summer than in winter with a marked seasonal difference within these four habitats (*p* < 0.05, Figure 3).

Sediment CH_4_ production rates varied from 35.61 to 181.29 μmol kg^–1^ d^–1^ with an average value of 109.94 ± 37.27 μmol kg^–1^ d^–1^ in summer and from 35.61 to 30.88 μmol kg^–1^ d^–1^ with an average value of 20.99 ± 6.16 μmol kg^–1^ d^–1^ in winter and varied significantly with seasonal variations (Figures 3A, B, *p* < 0.05 for all). The spatial distribution of sediment CH_4_ production rates showed a similar pattern in both summer and winter with increased from CM to SA-6 to SA-10 then to SA-14. The values in *C. malacensis* habitat were significantly higher than those in *S. alterniflora* habitats (*p* < 0.05, Figure 3A). The values in surface sediments (0-10 cm) were generally significantly higher than those in the subsurface sediments (10-20 cm) (*p* < 0.05 for all, excluding SA-14). Stepwise multiple regression analysis showed that sediment CH_4_ production rates were correlated positively to sediment bacterial abundances, TOC, and fungal gene abundances contents and correlated negatively with sulfide contents in summer, and were correlated positively to sediment bacterial abundances, and water contents and correlated negatively with sediment depth in winter (Table 1).

**TABLE 1:** The dependency of the CH_4_, CO_2_, N_2_O, CO_2_e, CH_4_%, CO_2_%, and N_2_O% in the multiple regression analyses using the stepwise method.

Dependent	Season	Factors in the model	Coefficients B	95% Confidence Interval for B		*Adjusted R* ^2^	Significance	D-W
				Lower Bound	Upper Bound			
CH_4_	Summer	Bacterial (copies g^–1^)	0.63	0.38	0.88	0.706	0.000	1.96
		TOC (mg C g^–1^)	0.75	0.50	1.00	0.794	0.000	
		Sulfide (mg S kg^–1^)	-0.60	-0.91	-0.30	0.890	0.001	
		Fungal (copies g^–1^)	0.28	0.10	0.47	0.924	0.005	
	Winter	Depth (cm)	-0.43	-0.69	-0.17	0.655	0.002	2.25
		Bacterial (copies g^–1^)	0.56	0.31	0.81	0.785	0.000	
		WC (%)	0.22	0.006	0.43	0.816	0.044	
CO_2_	Summer	TOC (mg C g^–1^)	0.48	0.32	0.64	0.725	0.000	1.76
		NO_2_^–^ (μg N kg^–1^)	0.25	0.05	0.45	0.894	0.016	
		Fungal (copies g^–1^)	0.24	0.05	0.42	0.916	0.017	
		TN (mg N g^–1^)	0.19	0.01	0.36	0.930	0.037	
	Winter	Bacterial (copies g^–1^)	0.61	0.36	0.85	0.885	0.000	1.52
		TOC (mg C g^–1^)	0.24	0.04	0.43	0.903	0.022	
		Fungal (copies g^–1^)	0.20	0.01	0.40	0.918	0.040	
N_2_O	Summer	Bacterial (copies g^–1^)	0.80	0.54	1.07	0.628	0.000	2.47
	Winter	Bacterial (copies g^–1^)	0.80	0.54	1.07	0.647	0.000	2.30
CO_2_e	Summer	Bacterial (copies g^–1^)	0.30	0.13	0.47	0.721	0.002	2.07
		TOC (mg C g^–1^)	0.57	0.42	0.72	0.888	0.000	
		Fungal (copies g^–1^)	0.25	0.09	0.41	0.930	0.005	
		EC (mS cm^–2^)	-0.13	-0.25	-0.01	0.942	0.034	
	Winter	Bacterial (copies g^–1^)	0.95	0.80	1.09	0.89	0.000	2.08
CH_4_%	Winter	TOC (mg C g^–1^)	-0.84	-1.10	-0.58	0.577	0.000	1.84
		Depth (cm)	-0.31	-0.57	-0.05	0.659	0.021	
CO_2_%	Winter	TOC (mg C g^–1^)	0.83	0.57	1.10	0.558	0.000	1.76
		Depth (cm)	0.33	0.06	0.59	0.646	0.019	
N_2_O%	Summer	Density (g mL^–1^)	0.43	0.07	0.79	0.401	0.023	1.82
		TOC:TN	-0.42	-0.78	-0.06	0.509	0.025	

D-W, Durbin-Watson; Tolerance > 0.2, VIF < 10. WC denote sediment water content.

Sediment CO_2_ production rates ranged from 3.02 to 12.42 mmol kg^–1^ d^–1^ with an average value of 7.71 ± 2.27 mmol kg^–1^ d^–1^ in summer and from 1.89 to 7.12 mmol kg^–1^ d^–1^ with an average value of 4.26 ± 1.40 mmol kg^–1^ d^–1^ in winter and varied significantly with seasonal variations (Figures 3C, D, *p* < 0.05 for all). Spatially, sediment CO_2_ production rates resembled the distribution patterns of CH_4_ production rates, namely they increased with the increasing invasion period of *S. alterniflora*. The annual mean rates among these four habitats varied spacially (*p* < 0.05 for all, excluding between SA-6 and SA-10 in 10-20 cm sediment layer). The rates at 0-10 cm sediment layer were significantly higher than those at 10-20 cm sediment layer both in summer and winter (*p* < 0.05). Moreover, sediment CO_2_ production rates correlated positively with sediment TOC, NO_2_^–^, fungal gene abundances, and TN in summer, and correlated positively with sediment bacterial gene abundances, TOC, and fungal gene abundances in winter when considering all sites (Table 1).

Sediment N_2_O production rates varied from 42.44 to 136.71 nmol kg^–1^ d^–1^ with an average value of 74.95 ± 22.62 mmol kg^–1^ d^–1^ in summer and from 15.81 to 66.49 nmol kg^–1^ d^–1^ with an average value of 37.14 ± 15.08 mmol kg^–1^ d^–1^ in winter and varied significantly with seasonal variations (Figures 3E, F, *p* < 0.05 for all). In general, sediment N_2_O production rates increased with the increasing invasion period of *S. alterniflora*. The rates in SA-10 and SA-14 were both significantly higher than those in CM in the surface sediments (0-10 cm) (*p* < 0.05), and the winter rates in SA-14 were significantly higher than those in CM and SA-6 in subsurface sediments (10-20 cm) (*p* < 0.05 for both). The rates at the surface (0-10 cm) were also significantly higher than those at the subsurface (10-20 cm) (*p* < 0.05 for all). Moreover, we found that sediment N_2_O production rates were correlated positively to sediment bacterial gene abundances in both summer and winter (Table 1).

The total GHG production rates were determined by converting the rates of CH_4_ and N_2_O production to CO_2_-equivalent (CO_2_e) rates and then adding the CO_2_e rates of CO_2_, CH_4_, and N_2_O together. Total GHGs rates ranged from 158.46 to 629.29 mg CO_2_ kg^–1^ d^–1^ with an average value of 389.54 ± 115.72 mmol kg^–1^ d^–1^ in summer and from 89.51 to 325.53 mg CO_2_ kg^–1^ d^–1^ with an average value of 197.08 ± 64.09 mmol kg^–1^ d^–1^ in winter, respectively and varied significantly with seasonal variations (Figures 3G, H, *p* < 0.05 for all). The total GHG production rates showed similar distribution patterns to those of sediment CH_4_ and CO_2_ production rates. Signiffcant spatial differences in the total GHG production rates in those habitats were observed (*p* < 0.05 for all; excluding between SA-6 and SA-10 in winter). The rates at the surface (0-10 cm) were significantly higher than those at subsurface (10-20 cm) both in summer and winter (*p* < 0.05). Additionally, the total GHG production rates were correlated positively with sediment sediment bacterial gene abundances, TOC, fungal gene abundances, and EC in summer, and correlated positively with sediment bacterial gene abundances in winter (Table 1).

**FIGURE 4:**
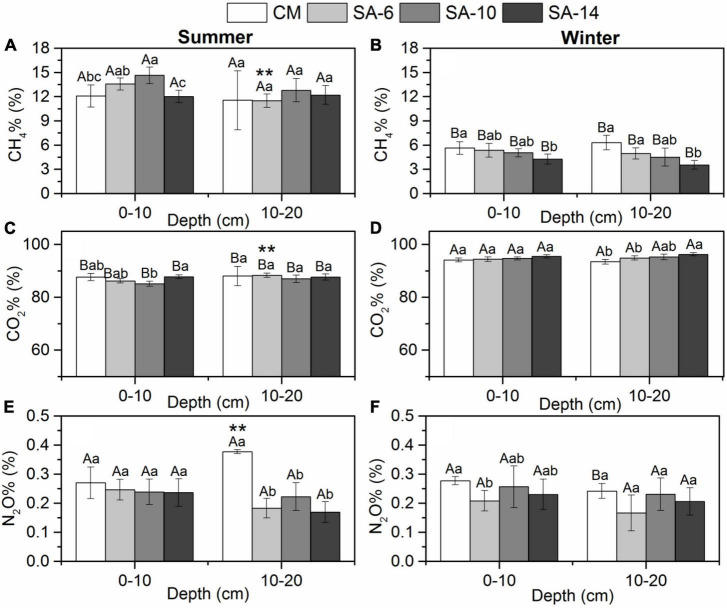
The effects of invasive *S. alternifolia* on sediment CH_4_% **(A,B)**, CO_2_% **(C,D)**, and N_2_O% **(E,F)** along four habitats (Mean ± SD). The CM denotes *C. malacensis* habitat, and the SA-6, SA-10, and SA-14 denote *S. alterniflora* habitats for 2–6, 6–10, and 10–14 years, respectively. Different lowercase letters are used to denote statistical differences (*p* < 0.05) across four habitats in the same depth, while upper case letters are used to denote significant differences (*p* < 0.05) between summer and winter in each habitat. ** are used to denote significant differences (*p* < 0.05) between surface and subsurface sediments.

The contribution of sediment CH_4_, CO_2_, and N_2_O production rates to total GHG production rates was expressed as CH_4_%, CO_2_%, and N_2_O%, respectively. There is no obvious change pattern in space in three values. CO_2_ was the predominant component of the total GHGs emissions (in CO_2_e) in both summer (87.22 ± 1.71%) and winter (94.83 ± 1.06%). The values were lower in summer than in winter with a marked seasonal difference within these four habitats (*p* < 0.05; Figures 4C, D). CO_2_% were correlated positively to sediment TOC and depth in winter (Table 1). CH_4_ represented 12.54 ± 1.71% of total GHGs emissions in summer and represented 4.95 ± 1.04% of total GHGs emissions in winter. CH_4_% was higher in summer than in winter with a significant seasonal variation (*p* < 0.05; Figures 4A, B). CH_4_% were correlated negatively to sediment TOC and depth in winter (Table 1). N_2_O was of minor importance to total GHGs emissions, which contributed 0.24 ± 0.07% and 0.23 ± 0.05% to total GHGs emissions in summer and winter, respectively. No seasonal variation in N_2_O% was observed in those four habitats in both two layers (*p* > 0.05; Figures 4E, F). N_2_O% were correlated positively to sediment bulk density and correlated negatively to sediment depth in summer (Table 1).

### 3.3 Sediment microbial characteristics

**FIGURE 5:**
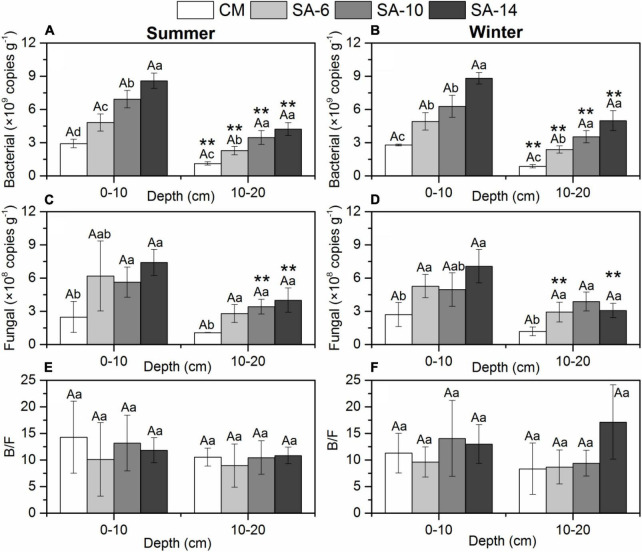
Sediment bacterial gene abundances **(A,B)**, fungal gene abundances **(C,D)**, and the ratio of B/F **(E,F)** along four habitats. Lowercase letters are used to indicate significant differences (*p* < 0.05) among different plant communities, while uppercase letters are used to show significant differences (*p* < 0.05) between summer and winter within each habitat. The error bars represent standard deviation. ** are used to denote significant differences (*p* < 0.05) between surface and subsurface sediments.

The bacterial 16S rRNA abundances ranged between 1.08–9.29 × 10^9^, and 0.72–9.36 × 10^9^ copies g^–1^ in summer and winter, respectively, showing no significant temporal differences (Figures 5A, B). Spatial distribution of sediment bacterial 16S rRNA abundances showed a similar pattern in both summer and winter with increased from CM to SA-6 to SA-10 then to SA-14. Significant spatial differences in bacterial 16S rRNA abundances observed among all habitats in summer and winter (*p* < 0.05). Also, the bacterial 16S rRNA abundances at 0-10 cm sediment layer were significantly higher than those at 10-20 cm sediment layer both in summer and winter (*p* < 0.05).

Fungal gene abundances were in a range of 1.03–9.37 × 10^8^, and 0.74–8.52 × 10^8^ copies g^–1^ in summer and winter, respectively, showing no significant temporal differences (Figures 5C, D). In general, this gene abundances increased with the increase in the invasion period of *S. alterniflora*. This values in CM habitat were generally significantly higher than those in *S. alterniflora* habitats (SA-6, SA-10, and SA-14) (*p* < 0.05 for all; Figures 5C, D). The fungal gene abundances in surface sediments (0-10 cm) were generally higher than those in subsurface sediments (10-20 cm). In addition, there is no significant seasonal and spatial difference in the ratio of bacterial gene abundances to fungal gene abundances (B/F) in our study (Figures 5E, F).

## 4 Discussion

Estuarine coastal wetland serve as transitional zones connecting terrestrial and marine ecosystems, which plays a significant role in the global C and N cycles and serve as an important source of GHGs emissions (Tan et al., 2020). The abundant vegetation found within wetlands can influence the production, consumption, and transport processes of GHGs, making it a key factor in regulating the “source” and “sink” functions of GHGs in wetland systems. This study focuses on the Minjiang Estuary as the research area, investigating the impact of different invasion durations of *S. alterniflora* on the potential GHGs production rates in sediments of the native salt marsh wetlands. We examined the patterns in GHGs production rates in surface and subsurface sediments across four habitats (CM, SA-6, SA-10, and SA-14) in the Minjiang Estuary. Seasonally, the production rates of GHGs were significantly higher in summer compared to winter (*p* < 0.05; Figure 3), and the seasonal fluctuations in these three rates were closely linked to changes in temperature. Spatially, sediment GHGs production rates increase from CM to SA-6 to SA-10 finally to SA-14. This spatial variation pattern in response to increasing sediment organic matter and microbial gene abundances (Figures 3, 5). Furthermore, these rates in the upper sediment layers (0-10 cm) were consistently observed to be significantly higher than those in the deeper layers (10-20 cm) (Figure 3). Thus, spatio-temporal variation characteristics of sediment GHGs production rates in the complex tidal flat wetlands are comprehensively influenced by various factors, including seasonal temperature fluctuations, vegetation types, and sediment depth. A comprehensive understanding of the environmental factors that drive the variability in sediment GHGs production rates in native *C. malacensis* invaded by *S. alterniflora* is crucial for evaluating the ecological impacts of this invasion.

Our findings provide important insights into the dynamics of sediment GHGs production rates in native *C. malacensis* wetlands invaded by *S. alterniflora*. Seasonally, sediment GHGs production rates were significantly higher in summer (32.97 ± 1.11°C) compared to winter (17.28 ± 0.47°C) in both surface and subsurface sediments among these habitats (*p* < 0.05 for all, Figure 3). Temperature has been identified as a significant factor that can impact GHGs emissions by influencing soil/sediment organic matter mineralization, chemical reaction rate, and gases diffusion (Baldock et al., 2012; Inglett et al., 2012). Numerous studies have demonstrated that increasing temperatures can enhance GHGs emissions from soil, potentially triggering a positive feedback loop to climate change (Davidson and Janssens, 2006; Bonnett et al., 2013; Li et al., 2020; Zhang et al., 2020). Also, GHGs emissions are predicted to raise the mean global temperature by 2.6–4.8°C by the end of the century (IPCC, 2014). Thus, seasonal temperature fluctuations as a key controlling factor driving seasonal variations in the GHGs production in estuarine and coastal sediments.

**FIGURE 6:**
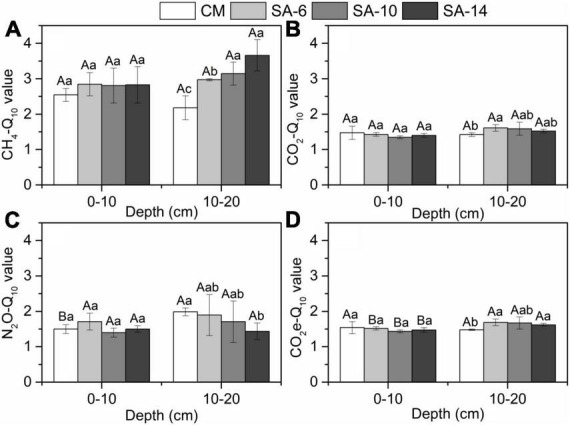
Variations in the temperature sensitivity (Q_10_) of sediment GHGs production rates **(A–D)** along four habitats. Different lower case letters indicate significant differences among these habitats, and upper case letters denote significant differences between the surface and subsurface sediments within each habitat. The errors represent standard deviation.

**FIGURE 7:**
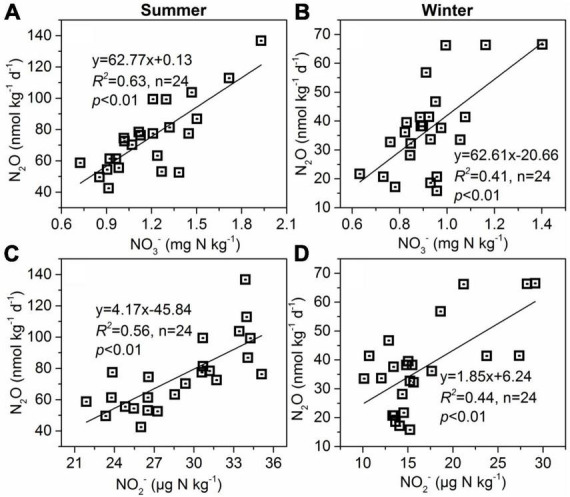
Relationships between sediment N_2_O production rates and sediment NO_3_^–^ **(A,B)** and NO_2_^–^ **(C,D)** contents in summer and winter.

*S. alterniflora* invasion can slightly increase the temperature sensitivities (Q_10_) of sediment CH_4_, CO_2_, and N_2_O production rates, but without significant differences (Figure 7). This is mainly attributed to the fact that *S. alterniflora* invasion can lead to an increase in sediment organic matter (substrates), but the degree of increase is not significant (Figure 2). Interestingly, we found that *S. alterniflora* invasion significantly increased the temperature sensitivity of total GHGs production rates in sub-surface sediments, but no significant difference was observed in surface sediments (Figure 6D). This finding is likely due to *S. alterniflora* having more developed root systems and a higher underground biomass than those of native *C. malacensis* (Jin et al., 2017). Developed root systems can effectively promote organic matter in deeper soil layers. Namely by changing the substrates concentrations required for GHGs production, it can affect the Q_10_ of GHGs production in the subsurface sediments. Nevertheless, there was no significant variation in the Q_10_ values of sediment GHGs production rates between surface and subsurface sediments (Figures 6A–D), suggesting that both surface and subsurface layers exhibited similar responses to increasing temperatures. Overall, *S. alterniflora* invasion has a limited impact on Q_10_ of sediment GHGs production rates, especially in surface sediments.

Meanwhile, temperature sensitivity of CH_4_ production rates calculated from seasonal temperature differences is significantly higher than that of the other two GHGs in the study (Figure 5A), and the CH_4_% in summer was obvious higher that in winter (Figures 4A, B). Considering that our study area is located in a subtropical monsoon climate zone, the salinity in the estuarine area is significantly lower in summer than in winter due to seasonal influences. CH_4_ emission is more responsive to salinity compared to the other two GHGs. Previous studies have generally shown a significant negative correlation between salinity and CH_4_ emission rates (Nyman and DeLaune, 1991; Marton et al., 2012; Neubauer, 2013). This is mainly due to the increase in the concentration of sulfate ions (SO_4_^2–^) available for sulfate-reducing bacteria with increasing salinity, leading to a shift from CH_4_ production processes to sulfate reduction processes in marsh wetlands, thereby reducing CH_4_ production rates (Sotomayor et al., 1994; Weston et al., 2006). In addition, sulfate-reducing bacteria can oxidize CH_4_, leading to a decrease in sediment CH_4_ emission (Devol and Ahmed, 1981; Iversen and Jørgensen, 1985). Thus, the seasonal variation characteristics of GHGs production rates, especially CH_4_, are also controlled by seasonal salinity change in estuarine and coastal sediments.

Our measured sediment GHGs production rates and organic matter (TOC and TN) showed a similar spatial distribution pattern (Figures 2, 3). Sediment GHGs production rates were larger in *S. alterniflora* than in *C. malacensis*, and the values increased with the invasion degree of *S. alterniflora*. We also found the sediment GHGs production rates were correlated positively to TOC (Table 1). It’s well believed that labile organic carbon is considered highly reactive indicator of GHGs emission in various ecosystems (Inubushi et al., 2003; Chen et al., 2010; Lin and Lin, 2022). The CO_2_ or CH_4_ release from sediment depend on mineralization, decomposition, or fermentation of organic carbon. In general, sediment organic matter concentrations in estuarine and coastal wetlands are closely linked to the growth of vegetation, and higher net primary production can increase their concentrations through litter and root inputs (Tong et al., 2011; Teng and Lin, 2024). It has been demonstrated that *S. alterniflora* exhibited higher above ground and root biomass in comparison to *C. malacensis* (Gao et al., 2017), which explained well the changes in TOC mentioned above. For N_2_O, the pathways of N_2_O production in tidal flat wetland sediments mainly include denitrification and nitrification processes. Previous studies have demonstrated that organic carbon plays a key role in controlling the heterotrophic denitrification process in sediment/soil from various ecosystems (Cheng et al., 2016; Huang et al., 2021; Zhang et al., 2023), organic carbon can function both as electron donors and as a source of energy for denitrifying bacteria (Shan et al., 2016). In anaerobic-dominated estuarine tidal flat sediments, the production of N_2_O may predominantly arise from denitrification processes. Thus, the organic carbon content in sediments shapes spatial variations in sediment N_2_O production rates by directly influencing heterotrophic denitrification processes.

In the vertical profile, we found that sediment TOC was higher in the top layer than in the subsurface in *C. malacensis*, while there were similar levels between these two depths in *S. alterniflora* (Figure 2). It is well known that sediment organic matter level in the surface layer is expected to be higher than that in the deeper layer due to the inputs of fallen twigs and leaves (Xia et al., 2021). This divergent outcome could be linked to variations in root systems across these habitats (Emery and Fulweiler, 2014). *S. alterniflora* has more extensive and developed root systems than *C. malacensis*, leading to a lack of significant decrease in organic matter contents at the subsurface layer (10-20 cm) in the *S. alterniflora*. Therefore, *S. alternifolia* invasion changed the vertical distribution characteristics of sediment organic matter. However, this alteration did not affect the vertical differences in GHGs production rates. We found that sediment GHGs production rates in surface sediments were significantly higher than those in subsurface sediments in both *C. malacensis* and *S. alterniflora* habitats. This maybe due to the fact that sediment GHGs production rates are mainly controlled by the microbial gene abundances in our study (Table 1), and the *S. alterniflora* invasion did not alter their vertical distribution characteristics (Figure 5).

The stepwise multiple regression analysis in the present study shows that bacterial 16S rRNA abundances in sediment was the key contributing factor to N_2_O production, accounting for 62.8% and 64.7% of the variation in summer and winter, respectively (Table 1). It is well known that microbial-driven nitrification and denitrification processes occurring in tidal marsh wetland sediments are the main sources of N_2_O production (Morse and Bernhardt, 2013). These two processes are primarily biological processes, thus the nitrifying and denitrifying bacteria are crucial in influencing N_2_O production in esturine and coastal wetland sediments (Wang et al., 2007). In addition, although sediment NH_4_^+^, NO_2_^–^, and NO_3_^–^ were not included in the model as a key factor controlling sediment N_2_O production rates through multiple regression analysis in our study (Table 1), linear correlation analysis showed significant correlations between N_2_O production rates and NO_2_^–^ and NO_3_^–^ (Figures 7A–D). Probably, denitrification was the major process for N_2_O production in these anaerobic environments in this study.

Previous research findings confirmed that *S. alterniflora* invasion generated higher CH_4_ emissions than those from *Phragmites australis*, *Scirpus mariqueter*, *Cyperus malacensis*, and bare tidal flats in estuarine and coastal wetlands, and it is consistent with our results (Table 2). *S. alterniflora*, as a C4 plant, can significantly increase sediment TOC, TN, MBC, humic substances and nutrient contents in tidal flat sediments. *S. alterniflora* invasion can increase soil CH_4_ emissions by changing sediment environmental conditions. Previous studies have demonstrated that under anaerobic conditions, CH_4_ can be transformed through the utilization of alternative electron acceptors, including sulfate, NO_3_^–^, Fe(III), Mn(III, IV), and humic substances (Bridgham et al., 2013). Also, *S. alterniflora* invasion inhibited the expression of CH_4_ oxidation-associated functional genes. In the *S. alterniflora* habitat, a greater relative abundance of type II methanotrophs and a lower relative abundance of type I methanotrophs were observed compared to the *Scirpus mariqueter* habitat (He et al., 2021). The significant increase in CH_4_ production was facilitated by the invasion of *S. alterniflora* should be taken seriously, given that the global warming potential of CH_4_ is about 28 times greater than that of CO_2_.

**TABLE 2:** Response of GHGs emission/production from to *S. alterniflora* in this study and other estuarine and coastal wetlands.

Study area	Ecosystems	CH_4_	CO_2_	N_2_O	References
Minjiang River Estuary, China	SA → *C. malacensis*	↑	Na	Na	(Tong et al., 2012)
Yancheng National Nature Reserve, Yancheng city, Jiangsu Province, China	SA → Bare mudflat	↑	Na	↓	(Yuan et al., 2014)
	SA → *S. salsa*	↑	Na	↓	(Yuan et al., 2014)
	SA → *P. australis*	↑	Na	↓	(Yuan et al., 2014)
Jiangsu Province, China.	SA → Bare mudflat	↑	Na	Na	(Xiang et al., 2015)
Zhangjiang Estuary, China	SA → Mangrove	↑	↓	Na	(Gao et al., 2018)
Chongming Island, China	SA → *P. australis*	↑	Na	Na	(Bu et al., 2019)
Chongming Island, China	SA → *S. mariqueter*	↑	Na	Na	(Bu et al., 2019)
Jiuduansha wetland, China	SA → Bare mudflat	↑	↑	Na	(He et al., 2021)
Jiuduansha wetland, China	SA → *P. australis*	↑	↓	Na	(He et al., 2021)
Jiuduansha wetland, China	SA → *S. mariqueter*	↑	Na	Na	(He et al., 2021)
Yangtze Estuary, China	SA → Bare mudflat	↑	Na	Na	(Yang et al., 2021)
Yangtze River estuary, China	SA → Bare mudflat	Na	Na	↓	(Yang et al., 2021)
**Minjiang Estuary, China**	**SA → *C. malacensis***	↑	↑	↑	**This study**

SA, *S. alterniflor*; *P. australis*, *Phragmites australis*; *S. mariqueter, Scirpus mariqueter*; *C. malacensis, Cyperus malacensis.* ↑ represents an increase and ↓ represents a decrease. – represents no significant changes, Na indicates data were not available.

For CO_2_, the invasion of *S. alterniflora*, a C4 plant, can enhance sediment CO_2_ production rates when invading C3 plants or bare mudflat. However, when invading mangrove wetlands, it may reduce the sediment CO_2_ production rates. Additionally, there are studies indicating that the invasion of *S. alterniflora* into *Phragmites australis* has no significant impact on CO_2_ emissions. From the perspective of GHGs emission reduction, if sediment organic carbon in ecosystems needs to be cycled and released into the atmosphere, we prefer it to be released in the form of CO_2_ rather than CH_4_.

It should be noted that our measured potential GHGs production rates may overestimate the GHGs emissions from wetlands and should only be considered as indicative. Future research should conduct a large number of field studies (a static closed chamber method) to obtain more accurate and realistic field survey data reflecting the emissions fluxes of GHGs in outdoor environments. In addition, salt marsh ecosystems are important blue carbon systems on Earth, but their sediments remain a significant source of GHGs emissions. It is essential to have a clearer understanding of the GHGs emissions from different media in salt marsh ecosystems to provide accurate foundational data support for carbon mitigation initiatives.

**FIGURE 8:**
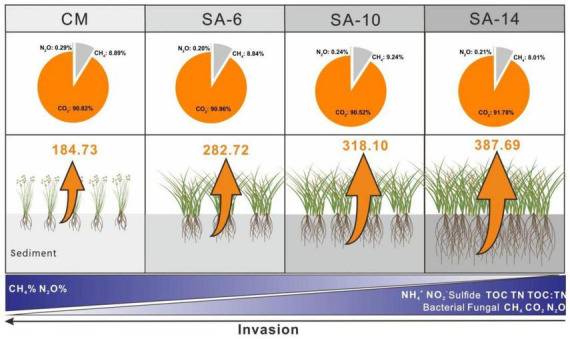
Sediment (0-20 cm) GHGs production rates and relative contributions along four types of plant community. The values are average for summer and winter. The orange arrows indicate the GHGs production rates, and the size of the arrow represents the magnitude of the rates. The CM denotes *C. malacensis* habitat, and the SA-6, SA-10, and SA-14 denote *S. alterniflora* habitats for 2–6 years, 6–10 years, and 10–14 years, respectively.

Our findings suggest that the presence of invasive *S. alterniflora* led to elevated organic matter levels in sediment within estuarine and coastal wetlands, potentially promoting C accumulation and storage (Figure 8). Nevertheless, the decomposition and mineralization potential of organic matter also rose with the degree of *S. alterniflora* invasion, leading to an acceleration in CH_4_, CO_2_, and N_2_O emissions that could impact climate change. Therefore, as the expansion of *S. alterniflora* continues and displaces native plants, there is a possibility of simultaneous increases in carbon accumulation and greenhouse gas emissions. Indeed, there are debates surrounding the ecological and environmental implications following the invasion of *S. alterniflora*, highlighting the need for further investigation. To gain a deeper insight into the dynamics of GHGs emissions in response to the invasive *S. alterniflora*, future studies should also focus on more about sediment microbial communities associated with GHGs production. Overall, our findings underscored the significance of *S. alterniflora* invasion in regulating GHGs production in coastal wetland sediments, providing essential data for estimating GHGs emissions and carbon sequestration in these intricate tidal wetland ecosystems.

## 5 Conclusion

Our study reported the variations in sediment physicochemical properties, GHGs production, and microbial gene abundances dynamics along invasion degrees of *S. alterniflora* in a subtropical estuarine wetland of China. Invasive *S. alterniflora* generally raised sediment organic matter and microbial gene abundances. Sediment GHGs production rates were also obviously influenced by *S. alterniflora* invasion, and their values increased with the degree of this exotic plant invasion. The GHGs production rates were characterized by higher values at the surface compared to the subsurface layer and by seasonal changes with higher values in summer than in winter. Temperature, organic matter and microbial gene abundances were found to be the key factors influencing the spatio-temporal variations of GHGs production rates. Overall, our findings showed the importance of *S. alterniflora* invasion in controlling GHGs production in coastal wetland sediments and provided fundamental data for estimating GHGs emissions and carbon sequestration in the complex tidal wetlands.

## Data availability statement

The data presented in the study are deposited in the figshare repository, it can be found online at https://figshare.com/authors/Xianbiao_Lin/18816934.

## Author contributions

DS: Conceptualization, Funding acquisition, Investigation, Project administration, Supervision, Writing–review and editing. YJ: Formal analysis, Funding acquisition, Investigation, Writing–original draft, Writing–review and editing. YS: Writing–review and editing. JH: Conceptualization, Formal analysis, Funding acquisition, Methodology, Supervision, Writing–review and editing. YD: Formal analysis, Writing–review and editing. YW: Writing–review and editing. HT: Writing–review and editing. JX: Writing–review and editing. DG: Investigation, Writing–review and editing. XL: Investigation, Writing–review and editing.
